# Silver Nanowires Modified with PEDOT: PSS and Graphene for Organic Light-Emitting Diodes Anode

**DOI:** 10.1038/srep45392

**Published:** 2017-03-28

**Authors:** Yilin Xu, Xiang Wei, Cong Wang, Jin Cao, Yigang Chen, Zhongquan Ma, Ying You, Jixiang Wan, Xiaohong Fang, Xiaoyuan Chen

**Affiliations:** 1 Thin Film Optoelectronic Technology Center, Shanghai Advanced Research Institute, Chinese Academy of Sciences, Shanghai 201210, China; 2 School of Materials Science and Engineering, Shanghai University, Shanghai 200444, China; 3 Key Laboratory of Advanced Display and System Application, Shanghai University, Shanghai 200072, China; 4 Department of Physics, Shanghai University, Shanghai 200444, China

## Abstract

Silver nanowires (AgNWs) networks are promising candidates for the replacement of indium tin oxide (ITO). However, the surface roughness of the AgNWs network is still too high for its application in optoelectronic devices. In this work, we have reduced the surface roughness of the AgNWs networks to 6.4 nm, compared to 33.9 nm of the as-deposited AgNWs network through the hot-pressing process, treatment with poly (3,4ethylenedioxythiophene)–poly (styrenesulfanate), and covered with graphene films. Using this method, we are able to produce AgNWs/PEDOT: PSS/SLG composite films with the transmittance and sheet resistance of 88.29% and 30 Ω/□, respectively. The OLEDs based on the AgNWs/PEDOT: PSS/SLG anodes are comparable to those based on ITO anodes.

Transparent conducting electrodes play an important role in large-area optoelectronic devices such as organic light-emitting diodes (OLED), solar cells and so on^1^,^2^,^3^,^4^,^5^. The prevailing material used in OLED electrodes is Tin-doped indium oxide (ITO) which has excellent photoelectric properties such as high optical transmittance and low sheet resistance^6^,^7^. However, ITO electrodes have some disadvantages such as high cost due to the shortage of indium and the diffusion of indium element into the light-emitting layer^8^. Therefore, it is necessary to explore new materials that can be applied in the transparent conductive anode in OLED. To replace ITO in transparent conducting electrodes, promising candidates include silver nanowires (AgNWs)^9^,^10^,^11^,^12^ and graphene^13^,^14^,^15^,^16^ which can provide both excellence electrical and optical performances, but they also have their own disadvantages. For example, the surface roughness of AgNWs network is relatively high due to the AgNWs junctions^17^, and it is well known that the resistance of single-layer graphene prepared by chemical vapor deposition (CVD) without doping is larger than 450 Ω/sq in general because of the existence of grain boundaries in polycrystalline graphene^18^,^19^. Q. Q Zhuo *et al*.^20^ reported that they used the direct production of graphene on substrates without transfer as the anode to fabricate OLEDs. Because the sheet resistance of N-doped graphene is still too high, it is difficult to obtain a good performance of the OLEDs only with graphene film as anode. In order to avoid these shortages, Saewon Kang *et al*.^21^ reported that they introduced a capillary printing technique to precisely control the nanowire alignment and the percolation behavior of the AgNWs network. The surface roughness (Rq) could be decreased from 33.9 nm to 15.6 nm. Moreover, they utilized the AgNWs/PEDOT: PSS composite transparent conductive film as OLED anodes to fabricate OLED devices with high luminous efficiency.

Although the nanowire entanglement issue for high-aspect-ratio AgNWs needs to be addressed, it shows great potential as the anodes of the OLEDs. Pei *et al*.^22^ recently reported that they used graphene oxide (GO) to wrap around and solder the AgNWs junctions to enhance the combination and reduce the square resistance. The composite membrane has excellent photoelectric properties, but the performance of the AgNWs/GO-based OLED devices is not satisfactory due to the high Rq of the composite film. In this work, we fabricated AgNWs/PEDOT: PSS/SLG (single-layer graphene) composite transparent conductive films with a low sheet resistance of 30 ± 5 Ω/sq and high optical transmittance above 87% in the visible spectrum. With the hot-press process, the surface roughness of the AgNWs network could be decreased from 33.9 nm to 9.5 nm, the surface wettability of AgNWs network was improve after the treatment of TiO_2_ sol coating. Furthermore, the surface roughness of the AgNWs network can be reduced to 6.4 nm with the cover of PEDOT: PSS. The AgNWs/PEDOT: PSS/Graphene composite transparent conductive films can be used as a great candidate to replace conventional ITO in large-area optoelectronic devices such as solar cells and touch panels.

## Results and Discussion

**Figure 1:**
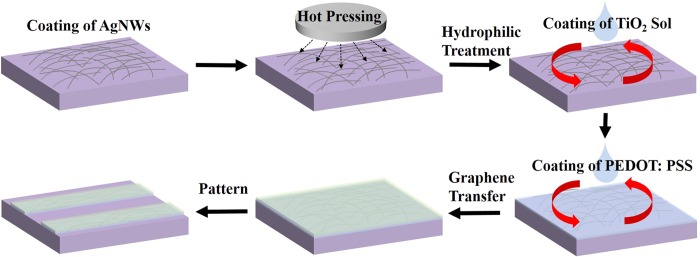
Schematic of the procedure to prepare the AgNWs/PEDOT: PSS/SLG composite film.

The procedure used to prepare the AgNWs/PEDOT: PSS/SLG composite transparent conductive film is schematically illustrated in Fig. 1.

### Characterization of the AgNWs films

**Figure 2:**
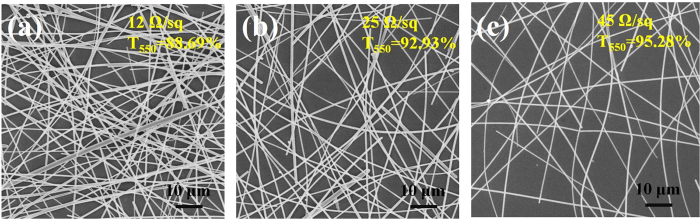
The SEM images of AgNWs networks with (**a**) 6 mg/ml, (**b**) 4.5 mg/ml, and (**c**) 3 mg/ml AgNWs solutions on quartz glass substrates.

In order to explore the relationship between the sheet resistance and transmittance of the AgNWs network, we have prepared a series of films using AgNWs solution with different concentrations of 6, 4.5 and 3 mg/ml scraping coated onto quartz glass substrates. Figure 2 displays the SEM of the AgNWs network with different AgNWs densities, we can see that the transparency and sheet resistance increase with decreasing AgNWs density due to the overlapping lines. Lee *et al*. also reported the similar AgNWs network^23^. Considering the balance between high transparency and low sheet resistance, we chose the AgNWs network prepared with 3 mg/ml AgNWs solution for further processing.

**Figure 3:**
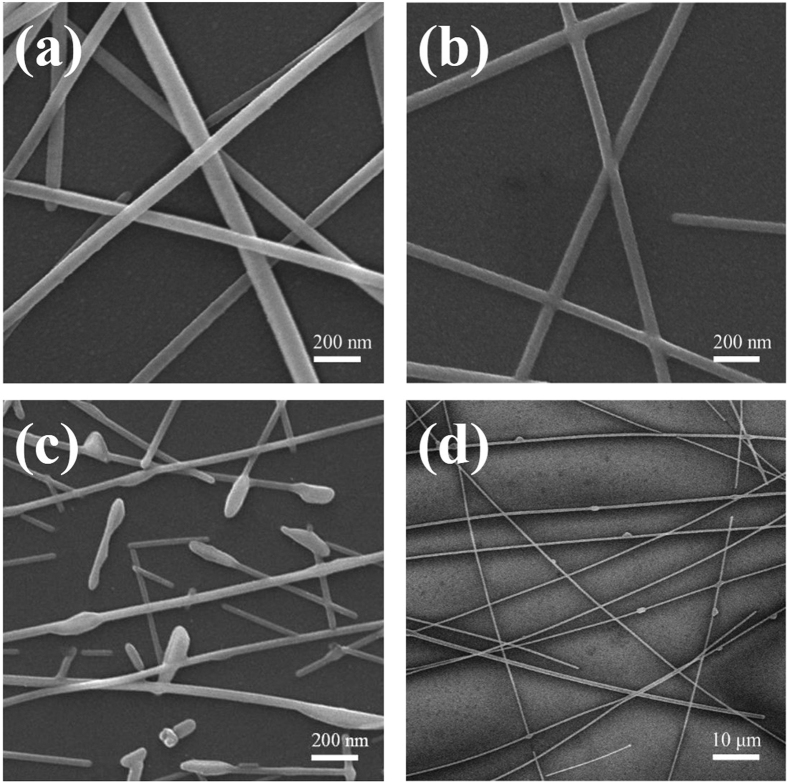
SEM images of the AgNWs networks with different hot-press temperatures of (**a**) 150 °C, (**b**) 200 °C, (**c**) 250 °C. (**d**) SEM image of the AgNWs/PEDOT: PSS film.

**Figure 4:**
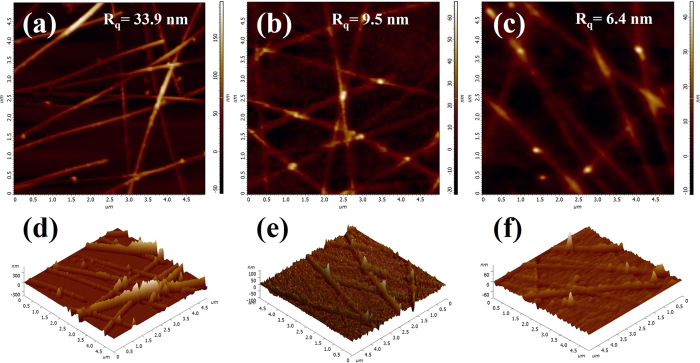
AFM images of the AgNWs networks with different hot-press temperatures of (**a**) 150 °C, (**b**) 200 °C. (**c**) AFM image of the AgNWs/PEDOT: PSS composite film after hot pressed at 200 °C. (**d**–**f**) for the (**a–c**) corresponding to the 3D display.

Although the AgNWs network has excellence conductivity and transparency comparable to ITO, its high surface roughness cannot fulfill the application requirements of OLEDs anodes. Thus, we use the hot-press process to reduce the surface roughness. The surface morphology of the AgNWs networks on the quartz glass substrate was measured by SEM and AFM, as shown in Figs 3 and 4. After hot-pressed at 150 °C, the surface roughness of the AgNWs network on the glass is 33.9 nm, which is still too high to be suitable for OLEDs. The roughness of the AgNWs network decreased to 9.5 nm after the hot press process at 200 °C, the AgNWs at junctions change from overlap state to self-welding state, as shown in Fig. 3(b), which could be due to self-limited welding or via Rayleigh instability as suggested by Garnett *et al*.^24^. It has indicated that hot press at 200 °C can strongly enhanced surface smoothness of the AgNWs network, and a similar effect was observed by Sahin Coskun *et al*. for annealing treatment with AgNWs network^25^. Further increasing the hot press temperature to 250 °C, we found that fractures occurred at the junction of the AgNWs, as shown in Fig. 3(c), leading to a decrease of the number of junctions and current transport paths. It is well known that PEDOT: PSS with good photoelectric property and high work function (5.1 eV–5.2 eV) is usually used as a hole transporting layer in organic optoelectronic devices^26^. We used PEDOT: PSS to reduce the roughness of the composite films, and the surface roughness of the composite film was decreased to 6.4 nm, as shown in Fig. 4(c). Figure 4(d–f) show the 3D display corresponding to Fig. 4(a–c), and it can be more intuitive to see the change of the roughness. Song *et al*. reported that they improved the characteristics of the AgNWs network through a process of irradiating it with intense pulsed light (IPL)^27^. The surface roughness of the AgNWs/PEDOT: PSS composite film treated with IPL can reach 5.9 nm. However, it is similar with the AgNWs/PEDOT: PSS composite film treated with hot-press process.

### Hydrophilic control of the AgNWs films

**Figure 5:**
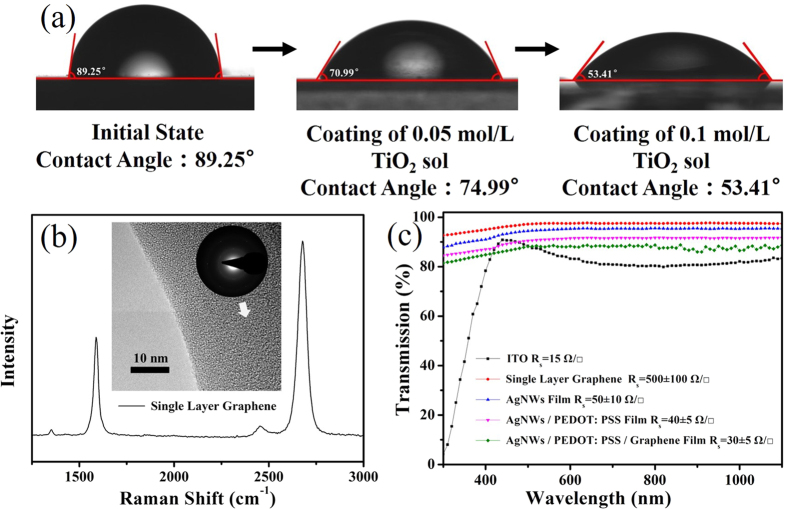
(**a**) Contact angles of the AgNWs network surfaces with different concentrations of the TiO_2_ sol treatment. (**b**) Raman spectrum and TEM image of a single-layer graphene. (**c**) Transmittance spectra of the ITO, single-layer graphene, AgNWs network, AgNWs/PEDOT: PSS, AgNWs/PEDOT: PSS/graphene and their sheet resistances.

It is difficult to cover the PEDOT: PSS aqueous dispersion on the AgNWs surface after hot press with good uniformity due to the poor hydrophilicity of the AgNWs network. A sol–gel TiO_2_ solutions were spin-coated on the AgNWs network to improve the surface wettability of the AgNWs films. Figure 5(a) shows the contact angles of the AgNWs networks after the sol–gel TiO_2_ solutions treatment with different concentrations. With the increasing concentration of the sol–gel TiO_2_ solutions, the contact angle was decreased from 89.25° to 53.41°, which suggests an improved hydrophily of the AgNWs network for the further deposition of PEDOT: PSS on the surface.

### Characterization of the AgNWs/PEDOT: PSS/SLG composite film

To further reduce the sheet resistance of the AgNWs/PEDOT: PSS films and improve the carrier mobility of the composite films, we use graphene films transferred to the surface of the composite film. Figure 5(b) shows the Raman spectrum of the graphene film to verify the number of the graphene layers. The sharp peaks at 1590 cm^−1^ and 2680 cm^−1^ are corresponding to the G band and 2D band of graphene, respectively^28^. From the TEM of the graphene film, we can see that it has only a single set of diffraction pattern for a regular hexagon. The intensity ratio of 2D band to G band is 1.9, and the weak peak (D band) at 1352 cm^−1^ shows a high crystalline quality of the single-layer graphene film^29^. Figure 5(c) shows the transmittance of the AgNWs network, SLG film, AgNWs/PEDOT: PSS film, AgNWs/PEDOT: PSS/SLG composite film and the reference ITO film as a function of wavelength in the range of 300–1100 nm. The transmittance of the AgNWs/PEDOT: PSS/SLG composite film and ITO at 550 nm is 88.29% and 85.56%, respectively. Particularly, the AgNWs/PEDOT: PSS/SLG composite film exhibits higher transmittance than that of the reference ITO in the range of 500–1100 nm. The sheet resistances of the AgNWs/PEDOT: PSS/SLG composite film and AgNWs/PEDOT: PSS composite film reach 30 Ω/□ and 40 Ω/□, slightly higher than that of ITO, which suggests that the AgNWs/PEDOT: PSS/SLG composite film is suitable for the application in OLEDs. The thicknesses of the AgNWs/PEDOT: PSS/SLG composite films and AgNWs/PEDOT: PSS composite films were in the range of 50–100 nm.

### Characterization of the OLEDs

**Figure 6:**
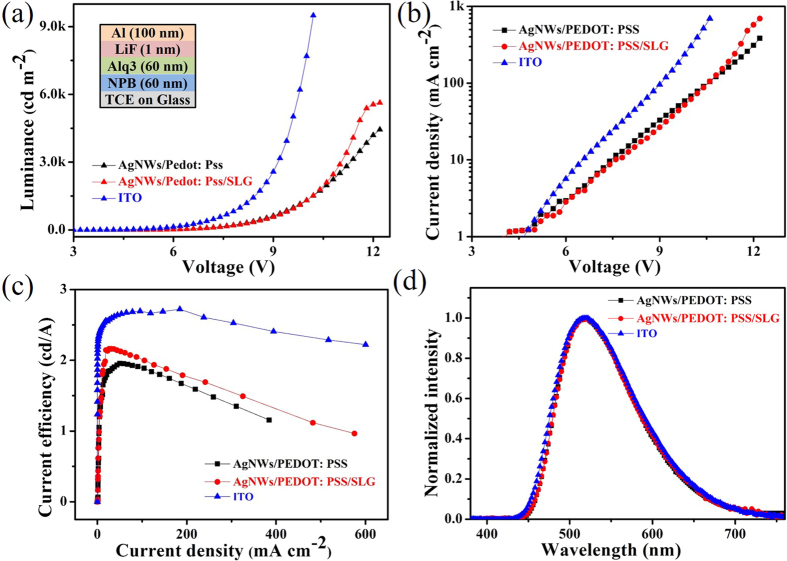
The structure of OLED and (**a**) Current density-voltage, (**b**) luminance-voltage characteristics, (**c**) Current efficiency-voltage and (**d**) electroluminescence spectra of the AgNWs/PEDOT: PSS and AgNWs/PEDOT: PSS/SLG and ITO based OLEDs.

The OLEDs based on the AgNWs/PEDOT: PSS/SLG and AgNWs/PEDOT: PSS composite film anodes were fabricated. The OLEDs based on the ITO anodes were also fabricated for comparison. The OLEDs have the structure of anode/NPB (60 nm)/Alq_3_ (60 nm)/LiF (1 nm)/Al (80 nm). Figure 6(a,b) show the current density–voltage characteristics and the luminance–voltage characteristics of the devices respectively, in which the current density and luminance at each specific drive voltage of the AgNWs/PEDOT: PSS/SLG and AgNWs/PEDOT: PSS composite films based devices exhibit a little lower value than those of the ITO based device. These devices based on the AgNWs/PEDOT: PSS and AgNWs/PEDOT: PSS/SLG films show a higher turn-on voltage of 4.4 V than those of the ITO based device (3.8 V). Figure 6(c) shows that the device based on the AgNWs/PEDOT: PSS anode exhibit a current efficiency (CE) of 1.64 cd/A measured at 200 mA/cm^2^, which were comparable to the CE of 1.78 cd/A at 200 mA/cm^2^ of the device based on the AgNWs/PEDOT: PSS/SLG anode. But the CE of the two devices are lower than those of the ITO based device which shows the CE of 2.68 cd/A measured at 200 mA/cm^2^. It is due to the higher sheet resistances of the AgNWs/PEDOT: PSS/SLG (~30 Ω/□) and AgNWs/PEDOT: PSS (~40 Ω/□) composite films than ITO (~15 Ω/□). Figure 6(d) shows the electroluminescence (EL) spectrum of these OLED devices at 10 V. The OLEDs based on AgNWs/PEDOT: PSS, AgNWs/PEDOT: PSS/SLG and ITO anode exhibited typical green electroluminescence from Alq3 with the CIE (Commission Internationale de l’Eclairage) coordinates of (0.32, 0.53), (0.32, 0.53) and (0.32, 0.51). It is obviously that the emitting light colors tend to be consistent and the influence of the different transmittances of the diverse anodes can be ignored.

## Conclusions

In conclusion, we have developed the AgNWs/PEDOT: PSS (~40 Ω/□, T_550nm_~91.04%) and AgNWs/PEDOT: PSS/SLG (~30 Ω/□, T_550nm_~88.29%) transparent conductive films with high transmittance, good conductivity and lower surface roughness. By a simple hot-pressing process, the roughness of the AgNWs network decreased from 33.9 nm to 9.5 nm. Further covered with PEDOT: PSS, which is readily used for the hole transport layer or hole injection layer in OLEDs, reduced the surface roughness to 6.4 nm. We have also demonstrated that the AgNWs network can be used as the transparent and conductive anodes in OLEDs. The performance of the device based on the AgNWs/PEDOT: PSS/SLG films is higher than the device based on the AgNWs/PEDOT: PSS films due to the contribution of graphene. These results suggest that the AgNWs/PEDOT: PSS/SLG film is a promising candidate as the transparent conductive electrode in optoelectronic devices.

## Experimental Procedures

### Preparation of AgNWs/PEDOT: PSS/SLG composite films

First, a quartz glass substrate (23 × 23 mm^2^) was cleaned with de-ionized water, acetone, and isopropanol.

### Preparation of the AgNWs network

The AgNWs (50 nm in diameter and 100–200 μm in length, XFNANO, China) dispersed in isopropyl alcohol with different concentrations was scraping coated onto the quartz glass substrate and annealed at 120 °C for 10 min before hot-pressing.

### Hot-press process

The sample was placed on the bearing platform of the imprinting equipment (NC-AX1401, Nano Carve), which was covered with a PI film (25 μm). The pressure of 0.3–1 MPa was applied between the top substrate and the PI film in the imprinting chamber with different heating temperatures to achieve non-contact hot-pressing.

### Hydrophilic treatment

The TiO_2_ solution was prepared by an ordinary method^30^. TiO_2_ sol–gel solution was spin-coated on the AgNWs network for 30 s at 6000 rpm to make it hydrophilic and annealed at 150 °C for 10 min. Then PEDOT: PSS (Clevios PH 1000, Heraeus) was spin-coated on the AgNWs network.

### Graphene growth and transfer

The SLG films were synthesized by CVD on a 25 μm polishing copper foil (99.999%, Alpha). Before growth, the foils were annealed at 1000 °C in H_2_ atmosphere for 5 min. Then, the source gas CH_4_ was infused with a flow rate of 0.5–3 sccm while keeping the same temperature for 3–10 min. Finally, the copper foils were rapidly cooled to room temperature. The graphene films were transferred to the surface of the AgNWs/PEDOT: PSS film to form the AgNWs/PEDOT: PSS/SLG composite film by a typical method^26^.

### Fabrication and measurement of OLEDs

The AgNWs/PEDOT: PSS/SLG composite film based anodes were patterned by photolithography technology and etching. OLED devices were fabricated with a structure of AgNWs/PEDOT: PSS/SLG/N, N ′- diphenyl - N, N′ - bis (1 - naphthyl) - (1, 1 ′- biphenyl) - 4, 4′ - diamin (NPB) (60 nm)/tris-(8-hydroxyquinoline) aluminium (Alq_3_) (60 nm)/LiF (1 nm)/Al (80 nm), where NPB was used as the hole-transporting material, Alq_3_ was used as both the host material and the electron-transporting material, and LiF (lithium fluoride) and Al were used as the electron-injecting material and the cathode material respectively.

To characterize the composite films, field-emission scanning electron microscope (SEM, FEI Quanta 600), transmission electron microscope, and atomic force microscope (NT-MDT) were used to examine the surface morphology. Raman measurements were performed using Thermo Scientific DXR Raman microscope spectrometer with a laser wavelength of 532 nm at room temperature. For sheet resistance measurements, we used a semiconductor analyzer (Agilent, B1500A) combined with a four-probe station (CASCADE, alessi REL-4800). The optical transmittance in the wavelength range of 300–1100 nm was obtained by a PV Measurements QEX10. The current–voltage (I–V) characteristics of the fabricated OLEDs were measured with an experimental set-up including a Keithley 2400 source meter. A spectroradiometer (PR750) was also employed to measure the electroluminescence spectrum of the 3 × 3 mm^2^ emitting area of the devices. The reference OLEDs with the same layer structures, except the AgNWs/PEDOT: PSS/SLG composite film was replaced by a conventional ITO layer (15 Ω/sq), were also fabricated for comparison.

## Additional Information

**How to cite this article**: Xu, Y. *et al*. Silver Nanowires Modified with PEDOT: PSS and Graphene for Organic Light-Emitting Diodes Anode. *Sci. Rep.* **7**, 45392; doi: 10.1038/srep45392 (2017).

**Publisher's note:** Springer Nature remains neutral with regard to jurisdictional claims in published maps and institutional affiliations.
